# Predominant miRNAs in Animal-Source Foods and Bioinformatic Analysis

**DOI:** 10.3390/cimb48020237

**Published:** 2026-02-23

**Authors:** Olubukunmi Amos Ilori, Giuseppe De Santis, Roberto Cannataro, Paola Tucci, Erika Cione

**Affiliations:** 1 Department of Pharmacy, Health and Nutritional Sciences, University of Calabria, 87036 Rende, Italy; olubukunmiamos.ilori@unical.it (O.A.I.); dsngpp99t11b774s@studenti.unical.it (G.D.S.); paola.tucci@unical.it (P.T.); erika.cione@unical.it (E.C.); 2 Galascreen Laboratories, University of Calabria, 87036 Rende, Italy; 3 Research Division, Dynamical Business & Science Society, DBSS International SAS, Bogota 110311, Colombia

**Keywords:** miRNAs, animal-source food, bioinformatics

## Abstract

The recognition of microRNAs as components of animal-source foods (ASFs) with epigenetic characteristics and regulation has spurred research in an interesting direction, particularly in understanding their microRNAs (miRNAs) fraction. Thus, a constant supply of them through food intake, with equally conserved targets, may facilitate their accumulation in tissues rich in their targets. Here, we consider the potentially dominant miRNAs in animal-source foods (ASFs) documented in the literature, identified through a frequency-weighted ordinal recurrence approach. *let*-7d-5p, miR-101-3p, and miR-133b consistently showed dominant rankings in a product-specific manner in lean meat. In meat fat, let-7i-5p, miR-30c-5p, and miR-23a-3p were highly ranked. Among various types of meat offal, miR-145-5p, miR-92-5p, and miR-24-3p emerged as the predominant miRNAs. Similarly, in dairy products, miR-200a-3p, miR-200c-3p, miR-223-3p, miR-25-3p, miR-29a-3p, and miR-29b-3p were recurrently dominant, whereas miR-17-5p, miR-184, miR-30e-5p, and miR-92b-3p showed a comparable prevalence in seafood. Even though bioinformatic approaches suggest miRNAs from raw ASFs showed major enrichment of processes and pathways culminating in epithelial barrier integrity modulation, such putative functions tend to be equally enriched by predicted targets of the miRNAs in processed products. Product-specific highly ranked miRNAs from food categories stipulate possible preferential enrichment in contexts of cell–cell adhesion, cytoskeletal dynamics, and inflammatory control by meat (lean, fat, offal), immune homeostasis by dairy, and neural signalling by seafood, providing hypotheses for future functional studies. However, a limited understanding of their stability during gastrointestinal transit may present a more immediate limitation to their potential translational applicability.

## 1. Introduction

Despite recommendations and clamour for increased consumption of plant-based foods and decreased animal-sourced foods to curtail the challenges facing sustainable food systems [1], global consumption of food products of animal origin, such as meat, poultry, dairy, and seafood, continues to grow, with the European Union being the consistently largest market [2,3]. Although there are considerations that most countries are reaching peak meat consumption [4], increases in incomes in low- and middle-income countries, coupled with urbanisation, support projections of a continual global increase in the ratio of animal-to-plant-sourced foods [2], as significant global differences in meat, dairy, and poultry consumption were observed for urban compared to rural areas [3]. Nutritional considerations also promote this; as per unit weight, foods of animal origin tend to be richer sources of the six nutrients and offer more energy [5]. Indications from recent studies suggest that such foods could offer more by exerting epigenetic regulation and post-transcriptional activities through their composition of bioactive nucleic acids [6,7]. A particular one of interest is the miRNA fraction.

miRNAs are short, non-coding RNA molecules distributed over a range of 18 to 25 nucleotides, which is approximately the length of a standard PCR primer and comprise about 0.01% of the total RNA typically extracted from a sample [8]. Many miRNAs originate from DNA sequences and are transcribed into primary miRNAs, which are then processed into precursor and mature miRNAs. Usually, miRNAs bind to target mRNAs’ 3′ untranslated region (UTR) to cause translational suppression and mRNA destruction [9]. These activities translate into a variety of biological processes accompanying development, metabolism, immunity, and even infection [10,11]. Identifying plant miRNA in animal tissues during the last decade, which suggested cross-kingdom transference, provided proof of their resistance to gastrointestinal digestion [12]. Since then, many investigations have gone on to ascribe this to the presence of protective structures, extracellular vesicles (EVs), or exosomes that shuttle these nucleic cargoes [13,14]. Their altered expressions are potential markers of diseased conditions, and experimental conditions often employ their overexpression or underexpression to produce predicted changes. Hence, constant supply through food intake, especially of animal sources, could provide a means of their accumulation. Being in the animal kingdom with equally conserved targets [15], their accumulation at a target site could produce biological effects [16]. Hence, livestock is seen as walking mounds of another set of interesting, potentially bioactive components. Whether the level obtained through oral ingestion is enough to produce effects will be a discussion beyond the scope of this work. However, there is a need for a unified view of the most abundant miRNAs that animal-sourced foods offer and their potential targets. Given that the mere presence of miRNAs is insufficient to exert biological effects, which are observed only when a certain concentration is reached [17], only the 10 most abundantly expressed miRNAs in such foods are considered in this paper.

## 2. Materials and Methods

### 2.1. Paper Selection

**Figure 1:**
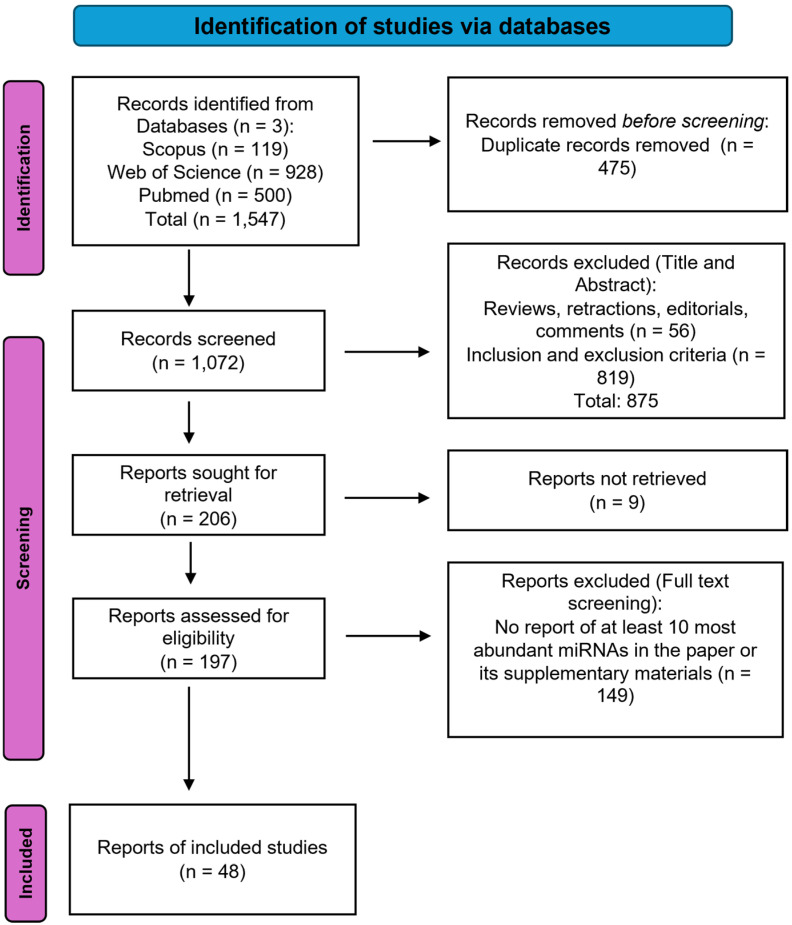
PRISMA flow chart for study selection.

Three databases (Scopus, Web of Science, and PubMed) were searched to identify studies reporting miRNA presence in edible components of animal tissues as of 1 January 2026. First, we defined “edible components” for paper selection as “widely consumed components or tissues originating from mature, uninfected animals generally considered as food”. We employed the following query: (mirna* OR microrna*) AND (meat* OR cow* OR cattle OR pork OR pig AND sheep OR goat* OR buffalo* OR rabbit* OR poultry OR egg* OR dairy OR milk OR yoghurt OR cheese* OR seafood OR crab OR prawn OR fish*) AND (food* OR diet*); excluding non-English papers, reviews, editorials, letters, conference papers. The selection of relevant studies followed PRISMA guidelines (Figure 1) [18]. Studies are included if they reported miRNA expression profiles derived from edible animal-source tissues or products intended for human consumption (e.g., muscle tissue, milk, adipose tissue, edible offals); examined healthy animals or included a healthy control group from which miRNA rankings could be extracted; provided sufficient methodological detail regarding tissue collection and RNA isolation procedures (e.g., description of tissue source, preservation method, and RNA extraction protocol); and reported either normalized expression values or explicit ordinal rankings of miRNAs. On the other hand, studies not reporting miRNA expression in primary animal tissues, investigating non-edible tissues or anatomical components not typically consumed as food, examining animals not commonly used for human consumption, focusing exclusively on infected, diseased, experimentally treated, fetal, embryonic, or pre-weaning animals, reporting miRNA expression exclusively from immortalised or non-primary cell cultures, and lacking sufficient methodological description of tissue sampling or RNA isolation procedures were excluded. Due to the heterogeneity of sample source and observational molecular profiling studies included in this paper, methodological quality was assessed by adapting the Joanna Briggs Institute (JBI) critical appraisal and Systematic Omics Analysis Review (SOAR) checklists [19,20]. This was structured into four components (sample quality, laboratory methodology, data and statistics, and reporting and reproducibility) to include molecular-specific criteria, which addressed healthy animal sample source, RNA quality control, sequencing/microarray validation, ranking method elaboration, and data reproducibility. Two authors independently performed the assessment, and disagreements, where present, were resolved by a third reviewer.

### 2.2. Target Prediction and Functional Analysis

Studies reporting at least the ten most abundant miRNAs in the paper body or providing supplementary information required to obtain such were considered. With the heterogeneity in sequencing platforms, normalisation strategies, and reporting formats across studies, the direct quantitative comparison of expression values was not feasible. Hence, a cumulative ordinal ranking or frequency-weighted ordinal recurrence approach was applied, whereby miRNAs received scores based on their within-study rank position and cumulative recurrence across independent studies, allowing the identification of miRNAs based on their consistent predominance in a food type from multiple studies. Specifically, the top ten miRNAs were assigned descending scores from 10, i.e., highest rank, to 1, i.e., tenth rank (a Borda-type rank), the summation of which was obtained for each product from multiple studies and used to finally rank the miRNAs, with each study having equal weights. This approach allows the evaluation of cross-study recurrence of high relative abundance rather than absolute or normalised quantitative equivalence, and it was also applied when combining multiple food types into a group (e.g., bovine breast, beef sirloin, lean pork tissue, etc., into lean meat) (Table S1). Only the highly conserved miRNAs [21] from them were included in the target prediction. Prediction of validated targets was performed using the multiMiR (v 1.32.0) [22], while the gene ontology (GO), KEGG, and Reactome Pathway analyses were undertaken using clusterProfiler (v 4.18.4) [23], ReactomePA (v 1.54.0) [24], and accompanying packages on RStudio (v 2023.06). The interaction between the top 100 most targeted proteins (by ≥40 of the miRNAs) was visualised using the STRING tool (https://string-db.org/, accessed on 9 January 2026) [25]. Additional plots for visualisation were generated using the SRPlot tool [26] and RStudio (v 2023.06).

## 3. Results and Discussion

### 3.1. Predominant miRNAs in Animal-Source Foods

We identified 48 studies that reported the top 10 consistently highly ranked miRNAs in terms of expression in animal tissues considered widely consumed food products (Table 1 and Tables S2A–C). Depending on the tissue sample, they account for about 43% to 97.4% of the mapped miRNAs. These studies established 48 unique highly conserved miRNAs, whose homologs are present in humans. Of these, there are 46 in raw products (Table 2), while 26 are identified in different processed animal-sourced foods (Table 3) across meat, dairy, and seafood groups. However, each tissue exhibits a unique profile, even from the same animal source. Raw bovine milk accounts for most of the studies as a single food product, although more research has focused on meat as a group than on other groups under animal-sourced foods, resulting in more meat components being profiled. Processed products were comparatively less represented in the selected studies. Table S1 presents the ordinal rankings of the miRNA in each of the five food groups in this study.

The miR-10b-5p and miR-1-3p are the most expressed miRNAs in the muscle tissue of selected animals, with miR-1 consistently among the top two expressed miRNAs in cow, pig, and rabbit meat [27,28,29,30,31,32,33,34,35]. miR-133a-3p and miR-26a-5p are also consistently expressed, although to a lesser extent. Both are widely conserved and are recognised regulators of muscle cell differentiation and proliferation, whose abundance in the muscle tissue is essential to adequate muscle growth, even in other species whose meat is commonly consumed [36,37,38,39,40]. miR-378 is the only other miRNA consistently expressed predominantly in the afore-identified meats, owing to its role in myotube formation via skeletal muscle vascularisation promotion [41,42]. However, it is not widely conserved, although all three homologs of the miRNA have a perfect identity match and coverage with the human homolog. Recognising that these abundances respond to thermal treatment and moisture removal is important. Depending on the part of the meat concerned, one to six miRNAs did not retain their position as the ten most abundant ones after cooking grounded beef at 177 °C until no pink meat remained [28]. Meat drying also resulted in the loss of two to four miRNAs from the top ten predominant ones [28]. Cooked ground pork also retained only six of its predominant ten miRNAs [30,31]. Despite these, miR-1-3p remained the most abundant in lean meat after cooking [28,31].

The miRNA portion of animal offal exhibits a high expression of miR-143 [28,33,43,44,45,46,47]. However, miRNA representation in various parts is unique. While miR-143 is the major miRNA expressed in the rumen and intestines, miR-122 is more consistent in liver products. miR-143’s role in epithelial regeneration and smooth muscle tonicity [48,49] and miR-122′s contribution to lipid and cholesterol metabolism [50,51] warrant their abundance in these components of offal. miRNA expression in the meat fat of livestock shows more disparity; nevertheless, miR-26a-5p is abundantly expressed in the meat fat from three of the four animals identified in this study [31,52,53,54,55], being vital in controlling fat tissue overgrowth [56].

Although the breast portion of livestock is considered to be a lean meat cut, it is important to outline its individual profile, as it is the site of milk production, which is an interesting food product in dietary miRNA research. The miRNA profile of the breast portion indicates high miR-143, miR-148a-3p, miR-26a, and miR-21-5p [33,34,35]. The dominance of miR148a and miR-21-5p, however, translates into equally high expression in milk from all five major dairy milk sources [57,58,59,60,61,62,63,64,65,66,67,68,69,70,71,72,73,74,75,76]. Cow milk, undoubtedly, is the most scrutinised food in this regard. Considering the miRNA levels in multiple studies with different sampling methods and lactation time [57,58,59,60,61,62,63,64,65,66,67,68,72,73,74], miR-148a-3p is still the dominant miRNA in cow milk, with its expression not affected despite a decrease in the extracellular vesicle content of milk associated with the lactation period [77]. While miR-148a-3p is the most expressed in cow milk and goat milk, our consensus ranking here identified let-7a-5p as the most consistently predominantly expressed miRNA in dairy milk, even more than miR-148a, which is often seen as the major milk miRNA and is correlated to milk yield [78,79].

The abundance of let-7a-5p expression is documented beyond the milk’s raw state. let-7a-5p is among the top ten most consistent, predominant miRNAs in milk products that have been processed through different means, not limited to sonication, thermal processing, homogenisation, and drying to yield products such as ultrasonicated milk, skimmed milk powder, butter, and infant formula [66,75,76,80,81]. miR-148a-3p abundance, however, is notably susceptible to sonication [66]. Of the documented processed dairy products, skimmed powdered milk is the most similar to dairy milk in terms of abundant, broadly conserved miRNA expression (nine of the ten miRNAs), while butter and infant formula are the least (five of the ten miRNAs).

As with the meat muscle, miR-1 is the paramount miRNA in fish muscle, accounting for 22.5% of normalised miRNA reads [82]. Xia et al. [83] also found the dominant expression of miR-1 in fish muscle to be tissue-specific. On the other hand, miR-22, which is also a highly expressed miRNA, was found to be stably expressed in Japanese flounder, irrespective of tissue type or condition [84]. Other seafood may contribute significant levels of certain miRNA homologs to the diets. For instance, sea cucumber contains different forms of the miR-92a, which has >94% alignment identity to the *hsa* homolog [85].

### 3.2. Putative Targets of Predominant miRNAs in ASFs as a Whole

The gene ontology of the targets of broadly conserved miRNAs that are consistently highly ranked in raw foods of animal sources (Figure S1) identified numerous biological processes significantly enriched, which are components of core cellular homeostasis and proteostasis-related activities. The processes range from intracellular trafficking to protein targeting and post-translational regulation, coupled with tight control of cellular metabolic and catabolic balance. The molecular function makeup presents binding and regulatory activities involved in signal transduction, intracellular trafficking, gene expression control and cell–cell adhesion. Their roles in tissue architecture maintenance are further reinforced through their cellular localisation, which is predominantly in cell–matrix interfaces and dynamic cytoplasmic regions, such as focal adhesion, cell–substrate junctions, and cell leading edge. Taken together, these form an integrated network that combines cell–matrix interactions and intracellular trafficking with transcriptional, metabolic, and proteostatic control. Though the processed foods documented exhibited a lesser number of the miRNAs, this network is maintained in their targets, with more enrichment in components of cellular dynamics, adhesion, and motility, such as the lamellipodium [86,87,88,89,90,91].

**Figure 2:**
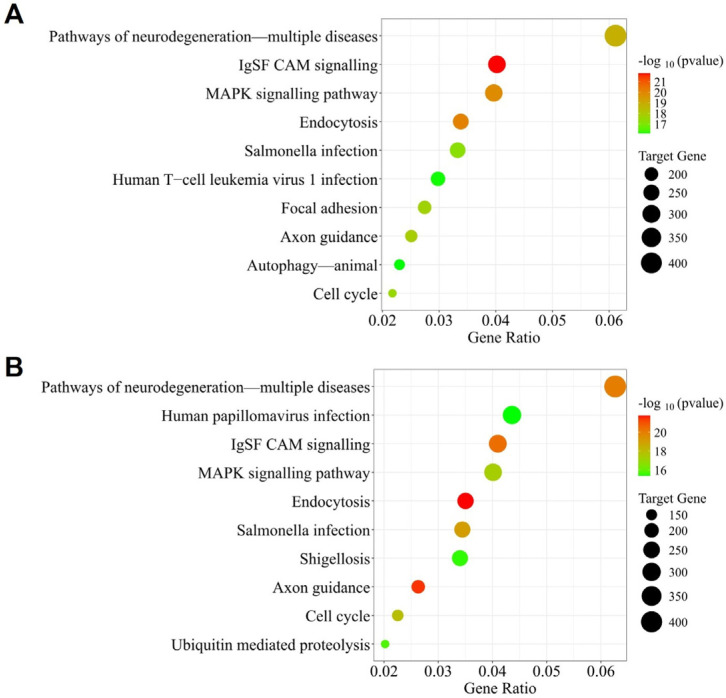
Potential pathways modulated by consistently highly ranked and broadly conserved miRNAs in foods of animal sources through KEGG pathway analysis of raw (**A**) and processed (**B**). Plotted using the SRPlot tool.

The KEGG enrichment (Figure 2A) of the miRNAs in raw products indicates a strong signal integration and stress-response regulatory network. This may manifest through cell-adhesion and guidance pathways (IgSF CAM signalling, focal adhesion), core signalling and fate-control pathways (MAPK signalling, cell cycle) and intracellular trafficking and stress-response pathways (endocytosis, autophagy) under normal and stress conditions, particularly in infection and neurodegenerative contexts. While there is an overlap in the adhesion-dependent signalling, intracellular trafficking, and cell fate decisions, the processed foods see a shift in these functions towards stress-induced regulation under infections and protein turnover (Figure 2B).

**Figure 3:**
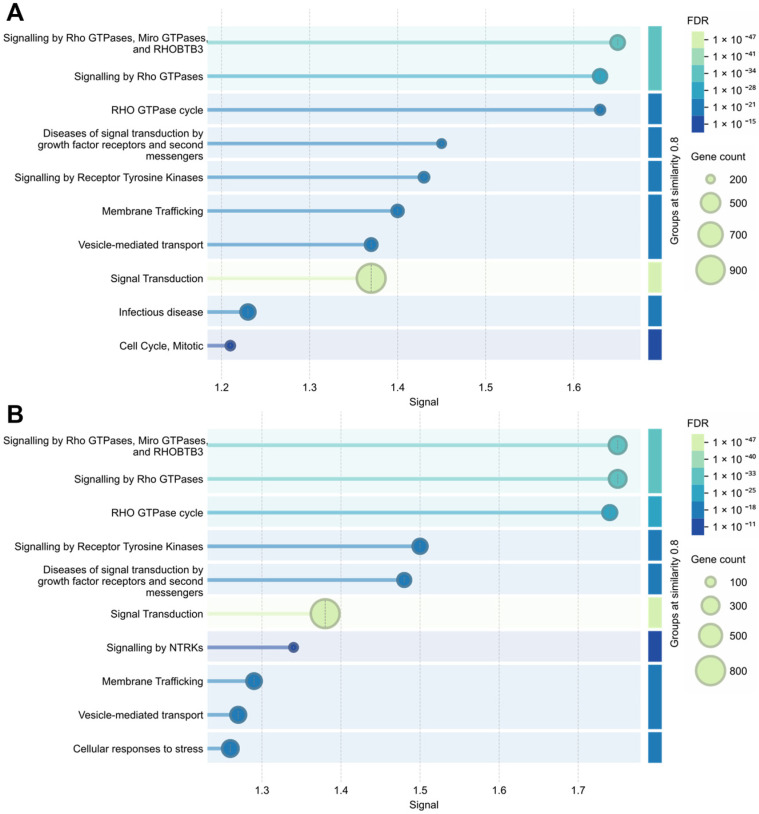
Potential pathways modulated by consistently highly ranked and broadly conserved miRNAs in foods of animal sources through Reactome pathway enrichment of raw (**A**) and processed (**B**). Plotted using the STRING tool.

The Reactome pathways of miRNAs from both the raw and the processed products are centred on small GTPase-driven signal integration, linking receptor-mediated signalling at the plasma membrane to cytoskeletal dynamics, intracellular trafficking, and downstream transcriptional responses (Figure 3A,B). The interaction between the IgSF CAM (immunoglobulin superfamily cell adhesion molecules) signalling and the Rho GTPase signalling has been fairly studied, and such interactions are vital in the regulation of the actin cytoskeleton. For instance, the netrin-1 attractive signalling is mediated through the IgSF CAM receptor Detected in Colorectal Cancer (DCC), which leads to the activation of intermediates (including PTK2 and FYN), resulting in the regulation of Rac1, CDC42, and RHOA, and in extension, the cytoskeleton and cell adhesion [92,93]. This can also be achieved via the Slit-Robo signalling [94]. Overall, the enrichment analyses provide for a possible conceptual modulation of adaptive cellular responses (such as cell motility and adhesion) by predominant, widely conserved miRNAs that are ASF-related via receptor tyrosine kinases (RTKs) to Rho GTPases [95], and to cytoskeletal dynamics and membrane trafficking [96], a conceptual framework that would benefit from the investigative input of future mechanistic studies.

**Figure 4:**
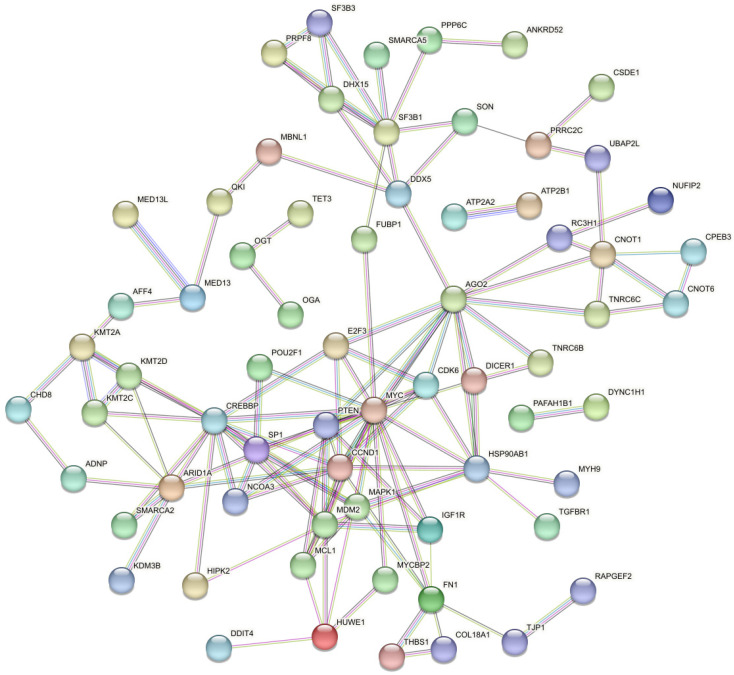
Protein–Protein Interaction (PPI) of the most targeted genes by >80% of consistently highly ranked and broadly conserved miRNAs in foods of animal sources (raw and processed). Performed using the STRING tool at high confidence (interaction score ≥ 0.7). Coloured nodes indicate query genes and edges indicate interactions between them.

Figure 4 depicts the interactions between the top 20% targeted proteins (of all the 48 miRNAs), identifying MYC (cellular myelocytomatosis oncogene) as the major hub protein (Table 4), which interacts with more than a quarter of the proteins in the model. It is a proto-oncogene transcription factor that binds DNA in a non-specific manner and activates the transcription of growth-related genes, which may influence epithelial integrity. Particularly, c-Myc has been demonstrated to modulate the intestinal barrier function through the activation of the E-cadherin promoter [97,98], as well as through c-Myc-regulated genes cyclin D1 (CCND1) and cyclin E [99]. Hence, it would not be surprising to find CCND1 as another highly targeted hub protein in the network.

**Table 1:** Ten consistently highly ranked miRNAs in different animal-sourced foods.

Food		10 Most-Abundant miRNAs		Method/Platform Employed	% of Mapped Fraction	Reference
		Shared ^1^	Selective ^2^			
**Raw Products**						
Meat	Lean	miR-148a-3p, miR-21-5p, miR-27b, miR-22-3p, miR-486-5p, miR-133a-3p, miR-30a-5p, miR-1-3p, miR-378, miR-206, miR-143-3p, miR-10b-5p, miR-26a-5p	Beef sirloin: miR-10a-5p	Illumina HiSeq 2000, HiSeq 2500, and HiSeq 4000	48.4–97.4	[27,28,29,30,32,33,34,35]
			Cow breast: miR-148d, miR-126-5p, miR-141			
			Goat breast: miR-186-5p, miR-181a-5p, miR-191-5p			
			Pork: let-7a-5p, let-7f			
			Rabbit: miR-26b-5p, miR-101-3p, let-7d-5p, let-7i-5p, miR-378d-5p			
	Fat	let-7a-5p, miR-125b, let-7f, let-7c, miR-26c, miR-143-3p, let-7i-5p, miR-26a-5p	Cow: let-7b, miR-2478, miR-126-3p, miR-2305, miR-1777b, miR-199a-3p	Agilent miRNA Microarray, Illumina HiSeq 2500 and HiSeq 2000, BGI/MGI DNA-SEQ	44.9–72.7	[52,53,54,55,87]
			Rabbit: miR-143-5p, miR-99a-5p, let-7i-3p, miR-99a-3p			
			Sheep: miR-133a-3p, miR-378-3p, miR-486-5p, miR-1-3p, miR-206, miR-30d, miR-30c			
			Buffalo: miR-451, miR-181a, miR-3600, miR-27b			
	Offal	miR-30a-5p, miR-26a-5p, miR-192, miR-27b, miR-148a-3p, miR-143-3p, miR-10b-5p, miR-99a-5p, miR-100-5p, miR-122-5p	Cow heart: miR-1-3p, miR-486-5p, miR-378a-3p, miR-30e-5p	Illumina HiSeq 2000, HiSeq 2000, HiSeq 2500, and NovaSeq 6000	52.6–95.3	[28,33,43,44,45,46,47,88]
			Cow liver: miR-26a, let-7f, miR-3600, miR-22-3p			
			Cow rumen and intestines: miR-10a-5p, miR-145, miR-24-3p, miR-21-5p			
			Rabbit liver: miR-122-3p, miR-99a-3p, miR-26c			
Dairy	Bovine colostrum	let-7a-5p, miR-30a-5p, miR-148a, let-7f, let-7b-5p, miR-26a, miR-181a, miR-22-3p, miR-1246, miR-21-5p		Illumina HiSeq 2500, 4000, and NovaSeqX	54.5–71	[72,73,89,90]
	Milk	miR-200c, miR-1246, miR-25, let-7f, miR-141, miR-30a-5p, miR-26a-5p, miR-200a, miR-148a-3p, miR-21-5p, let-7a-5p, let-7b-5p	Cow milk: miR-22-3p, miR-30d	Agilent Microarray, Affymetrix GeneChip Microarray; Illumina HiSeq 2000, HiSeq 2500, 4000, MiSeq 3000; NextSeq 500, NovaSeqX, GenomEast	58.6–95.7	[57,58,59,60,61,62,63,64,65,66,67,68,69,70,71,72,73,74,75,76]
			Buffalo milk: miR-11987, miR-2904			
			Goat milk: miR-223			
			Sheep milk: miR-29a, miR-16b, miR-26b			
			Camel milk: miR-29b, let-7c, miR-191, miR-92a-3p			
Seafood	Nile Tilapia muscle	Oni-miR-1, miR-206, miR-199, miR-22a, miR-214, miR-125b, miR-133a, miR-130c, miR-126, miR-17a		SOLiD Sequencing	61.1	[82]
	Pearl Oyster	Let-7-x, miR-100-x, miR-125-x, miR-184-y, miR-100-z, miR-99-x, miR-26-x, miR-30-x, miR-71-x, miR-27-y		Illumina HiSeq Xten	83.7	[91]
	Sea Cucumber	spu-miR-92b-3p, miR-184, efu-miR-92a, spu-miR-92a, miR-125-5p, miR-92b-3p_1, miR-2011, pmi-miR-2006-5p, spu-miR-252b, spu-miR-92b-3p_2		Illumina HiSeq 2500	83.8	[85]
**Processed products**						
Meat	Cooked lean tissue	miR-1-3p, miR-206, miR-378, miR-143-3p, miR-26a-5p, miR-133a-3p	Beef sirloin: miR-10b-5p, miR-30a-5p, miR-486-5p, miR-22-3p	Illumina HiSeq 2000 and HiSeq 2500	86–91	[28,31]
			Pork: miR-27b, miR-99a, miR-133b, miR-126-3p			
	Cooked fat tissue (pork)	miR-125b, miR-26a-5p, miR-143-3p, miR-27a, miR-99a-5p, miR-148a-3p, miR-23a, miR-21-5p, miR-126-3p, let-7a-5p		Illumina HiSeq 2500	43	[31]
	Cooked offal	miR-143-3p, miR-99a-5p, miR-30a-5p, miR-26a-5p	Beef heart: miR-1-3p, miR-10b-5p, miR-100-5p, miR-486-5p, miR-378a-3p, miR-27b-3p	Illumina HiSeq 2000 and HiSeq 2500	67–71	[28,31]
			Pork liver: miR-122, miR-451, let-7a-5p, miR-125b, miR-192, let-7f			
Dairy	Ultrasonicated milk	Bta-miR-320a, miR-122, let-7a-5p, miR-30d, miR-125b, miR-200c, let-7b, miR-21-5p, miR-30a-5p, let-7f		Illumina NextSeq 500	60	[66]
	Skimmed powder milk	Bta-let-7a-5p, let-7f, miR-30d, let-7b, miR-148a, miR-21-5p, miR-148d, miR-30a-5p, miR-200c, miR-26a		GenomEast	58.4	[76]
	Butter	Bta-miR-10b-5p, miR-22-3p, miR-148a-3p, miR-133a-3p, miR-30a-5p, miR-378-5p, miR-486-5p, miR-26a-5p, let-7a-5p, miR-143-3p		Illumina HiSeq 4000	~64	[80]
	Infant formula	Bta-let-7a-5p, let-7b, let-7f, miR-1246, miR-2887, miR-11975, let-7c, miR-11976, miR-200c, miR-2478 ^¶^, miR-11980 ^¶^		Illumina NovaSeqX	44.8	[75,81]

^1^ Shared indicates presence among the 10 consistently highly ranked in at least 2 of the food products within the food type. ^2^ Selective indicates the food product-specific, consistently highly ranked miRNAs within the food category. ^¶^ Ranked similarly.

**Table 2:** *Hsa* homologs of highly conserved predominant miRNAs identified by cross-study rank aggregation in raw animal-source foods.

miRNA	Animal-Derived Sources	miRNA	Animal-Derived Sources
let-7a-5p	Lean pork, meat fat (beef, rabbit), bovine colostrum, milk (cow, buffalo, goat, sheep, camel), pearl oyster	miR-192-5p	Offal (beef liver, beef rumen and intestines)
let-7b-5p	Beef fat, bovine colostrum, milk (cow, buffalo, goat, sheep, camel)	miR-199a-3p	Beef fat, tilapia fish
let-7c-5p	Meat fat (beef, buffalo), camel milk	miR-200a-3p	Milk (buffalo, goat, sheep)
let-7d-5p	Lean rabbit meat	miR-200c-3p	Milk (cow, camel)
let-7f-5p	Lean pork, meat fat (beef, buffalo), beef liver, bovine colostrum, milk (goat, camel)	miR-206	Lean meat (beef, pork, rabbit), sheep meat fat, tilapia fish
let-7i-5p	Rabbit meat fat	miR-21-5p	Lean meat (beef, goat), beef rumen and intestines, bovine colostrum, milk (cow, buffalo, goat, sheep)
miR-1-3p	Tilapia fish, lean meat (beef, pork, rabbit), sheep fat, beef heart	miR-22-3p	Lean meat (beef, goat), beef liver, bovine colostrum, cow milk, tilapia fish
miR-100-5p	Offal (beef heart, beef rumen and intestines, rabbit liver), pearl oyster	miR-223-3p	Goat milk
miR-101-3p	Lean rabbit meat	miR-24-3p	Beef rumen and intestines
miR-10a-5p	Lean beef, beef rumen and intestines	miR-25-3p	Milk (buffalo, camel)
miR-10b-5p	Lean meat (beef, goat, pork), offal (beef heart, beef rumen and intestines)	miR-26a-5p	Lean meat (beef, goat, pork), meat fat (rabbit, sheep, buffalo), offal (beef heart, beef liver, beef rumen and intestines), bovine colostrum, milk (cow, goat, sheep), pearl oyster
miR-122-5p	Offal (beef liver, rabbit liver)	miR-26b-5p	Lean rabbit meat, sheep milk
miR-125b-5p	Meat fat (beef, sheep), Tilapia fish, pearl oyster, sea cucumber	miR-27b-3p	Lean meat (beef, rabbit), buffalo meat fat, offal (beef liver, beef rumen and intestines), pearl oyster
miR-126-3p	Beef fat	miR-29a-3p	Sheep milk
miR-133a-3p	Lean meat (pork, rabbit), sheep fat, tilapia fish	miR-29b-3p	Camel milk
miR-141-3p	Lean beef, milk (cow, buffalo, goat)	miR-30a-5p	Lean meat (beef, goat), offal (beef heart, beef liver, beef rumen and intestines), bovine colostrum, milk (cow, goat, sheep)
miR-143-3p	Lean meat (beef, goat, pork), meat fat (rabbit, buffalo), offal (beef heart, beef liver, beef rumen and intestines)	miR-30c-5p	Sheep meat fat
miR-145-5p	Beef rumen and intestines	miR-30d-5p	Sheep meat fat, cow milk
miR-148a-3p	Lean meat (beef, goat), offal (beef liver, rabbit liver), bovine colostrum, milk (cow, buffalo, goat, sheep)	miR-30e-5p	Beef heart, pearl oyster
miR-17-5p	Tilapia fish	miR-451a	Buffalo meat fat
miR-181a-5p	Lean goat meat, buffalo meat fat, bovine colostrum	miR-92a-3p	Camel milk, sea cucumber
miR-184	Pearl oyster, sea cucumber	miR-92b-3p	Sea cucumber
miR-191-5p	Lean goat meat, camel milk	miR-99a-5p	Rabbit meat fat, offal (beef heart, rabbit liver), pearl oyster

**Table 3:** *Hsa* homologs of highly conserved predominant miRNAs identified by cross-study rank aggregation in processed animal-source foods.

miRNA	Animal-Derived Sources	miRNA	Animal-Derived Sources
let-7a-5p	Cooked pork fat, cooked pork liver, ultrasonicated milk, skimmed powder milk, butter, infant formula	miR-148a-3p	Cooked pork fat, skimmed powder milk, butter
let-7b-5p	Ultrasonicated milk, skimmed powder milk, infant formula	miR-192-5p	Cooked pork liver
let-7c-5p	Infant formula	miR-200c-3p	Ultrasonicated milk, skimmed powder milk, infant formula
let-7f-5p	Cooked pork liver, ultrasonicated milk, skimmed powder milk, infant formula	miR-206	Cooked lean meat (beef, pork)
miR-1-3p	Cooked lean meat (beef, pork), cooked beef heart	miR-21-5p	Cooked pork fat, ultrasonicated milk, skimmed powder milk
miR-100-5p	Cooked beef heart	miR-22-3p	Cooked lean beef, butter
miR-10b-5p	Cooked lean beef, cooked beef heart, butter	miR-23a-3p	Cooked pork fat
miR-122-5p	Cooked pork liver, ultrasonicated milk	miR-26a-5p	Cooked lean meat (beef, pork), cooked pork fat, cooked offal (beef heart, pork liver), skimmed powder milk, butter
miR-125b-5p	Cooked pork fat, cooked pork liver, ultrasonicated milk	miR-27b-3p	Cooked lean pork, cooked beef heart
miR-126-3p	Cooked lean beef, cooked pork fat	miR-30a-5p	Cooked lean beef, cooked offal (beef heart, pork liver), ultrasonicated milk, skimmed powder milk, butter
miR-133a-3p	Cooked lean meat (beef, pork), butter	miR-30d-5p	Ultrasonicated milk, skimmed powder milk
miR-133b	Cooked lean pork	miR-451a	Cooked pork liver
miR-143-3p	Cooked lean meat (beef, pork), cooked pork fat, cooked offal (beef heart, pork liver), butter	miR-99a-5p	Cooked lean pork, cooked pork fat, cooked offal (beef heart, pork liver)

**Table 4:** Node degrees of the components of the PPI of the most targeted genes by >80% broadly conserved miRNAs that ranked highly in animal-source foods.

Node	Degrees	Node	Degrees	Node	Degrees
MYC	18	KMT2A	5	COL18A1	2
CREBBP	13	DHX15	4	CPEB3	2
AGO2	12	DICER1	4	FUBP1	2
CCND1	12	IGF1R	4	HIPK2	2
MDM2	10	KMT2C	4	MBNL1	2
HSP90AB1	9	KMT2D	4	MYCBP2	2
PTEN	9	NCOA3	4	OGT	2
ARID1A	8	CNOT6	3	POU2F1	2
SF3B1	8	MED13	3	PPP6C	2
FN1	7	PRPF8	3	QKI	2
CDK6	6	PRRC2C	3	SMARCA2	2
CNOT1	6	RC3H1	3	THBS1	2
MAPK1	6	SF3B3	3	TJP1	2
MCL1	6	SON	3	TNRC6B	2
SP1	6	TNRC6C	3	UBAP2L	2
DDX5	5	ADNP	2		
E2F3	5	AFF4	2		
HUWE1	5	CHD8	2		

Listed targets are only those with at least a node degree of 2.

### 3.3. Selectively Predominant miRNAs in Animal-Source Foods and Their Potential Roles

**Figure 5:**
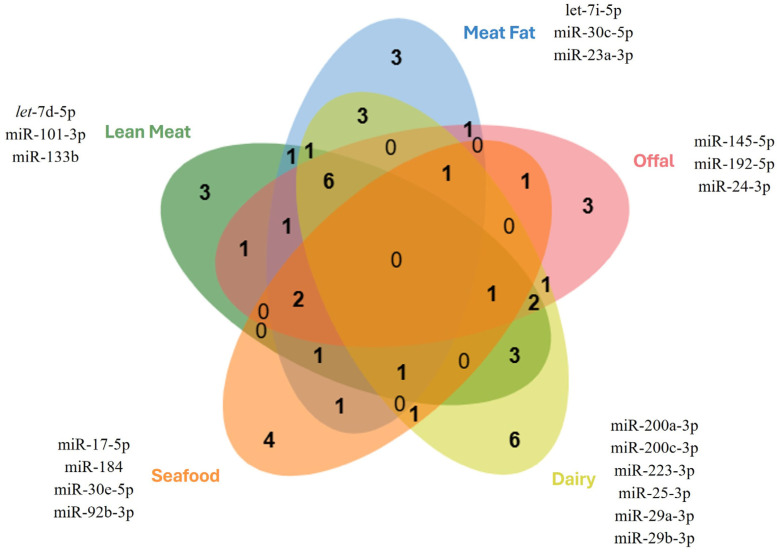
Food product-restricted highly ranked and widely conserved miRNAs in ASFs, indicating the selectively predominant miRNAs in lean meat, meat fat, meat offal, dairy, and seafood groups.

The categorisation of the documented foods (Table 2 and Table 3) into five groups reveals overlaps in the miRNAs present, leaving three, three, three, six, and four selectively predominant miRNAs that are widely conserved, respectively, in lean meat, meat fat, meat offal, dairy, and seafood (Figure 5).

Of these nineteen, only four remained abundant after processing: miR-133b (cooked lean pork), miR-23a-3p (cooked pork fat), miR-192-5p (cooked pork liver), and miR-200c-3p (ultrasonicated milk, skimmed powder milk, and infant formula). The miR-133b is considered a canonical myomiR [100] noted for its potency to act as a biomarker for tumour cells due to its consistent downregulation [101]. Similarly, it has the potential to regulate epithelial–mesenchymal transition (EMT) of epithelial cells, which contributes to epithelial integrity [102]. miR-23a-3p is an anti-inflammatory miRNA, with evidence of inflammatory regulation in different disease models, such as sepsis, diabetic kidney disease, and brain injury [103,104,105]. miR-192-5p is a potent promoter of epithelial integrity, particularly curtailing barrier dysfunction associated with inflammation [106]. It also does this through interaction with the TGF-β2 to modulate ECM components [107], while engaging the TGF-β1 in other contexts, such as through fibulin-2 in the brain [108]. On the other hand, miR-200c is a notable oncomiR, with documented evidence in different cancer types, promoting cancer development, tumour growth, invasion, and migration by targeting the Polycomb group (PcG) complex components [109].

Whether these miRNAs, if assumed through the diet, can produce such actions in vivo is yet to be demonstrated experimentally, since their digestive stability and bioavailability have not been recorded. However, a closely related variant of miR-133b, which is abundant in lean meat, meat fat, and fish (miR-133a-3p), was shown to survive gastrointestinal digestion in buffalo milk [110]. Similarly, miR-23a-3p is considered to be intrinsically stable [111]. This culminates in the potential of their survival post-digestion. Given their abundance in the food, if they survive digestion and such a level of concentration is maintained at the site of absorption, there is increased potential for evoking a biological response, since their function is not only dependent on their presence but also on their level of concentration after digestion [17]. In addition, a number of the miRNAs abundantly expressed in cooked or processed foods have been shown to be digestion-stable. These include miR-148a-3p, miR-30a-5p, miR-26a-5p, miR-22-3p, miR-125b-5p, and miR-27b-3p [17,110,112]. This survival is attributed to their encapsulation in extracellular vesicles.

To understand the specific potential biological functions of predicted targets of the miRNAs identified in each food group (Table S3), the most significant targets (components of their top ten most enriched KEGG pathways) of the selectively predominant miRNAs (cross-study consistent high ranking) in each group, as categorised in Figure 5, were clustered using the default parameters of the κ-means clustering component of the STRING tool to identify their three most important functions. It is important to note that the following mechanistic interpretations are purely speculative, being derived from computational target prediction and pathway enrichment analyses and should therefore be considered hypothesis-generating. These speculations are heavily dependent on whether the miRNAs are present in biologically sufficient quantities following dietary exposure, and do not demonstrate in vivo biological activity of dietary miRNAs; consequently, experimental validation will be required to determine physiological relevance.

#### 3.3.1. Lean Meat

**Figure 6:**
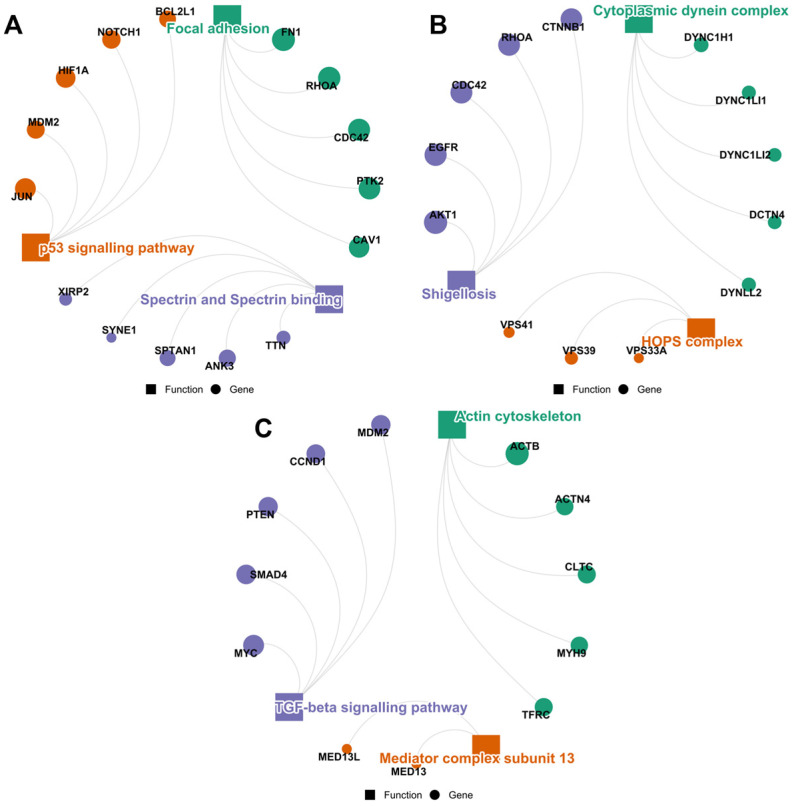
PPI cluster circular plots of the concordant/shared targets of meat-restricted highly ranked and widely conserved miRNAs, indicating the hub proteins (top five) and most significant functions (clusters) potentially modulated by (**A**) lean meat, (**B**) meat fat, and (**C**) meat offal. Circle diameter is directly proportional to the node degrees.

The lean meat exhibited cross-study recurrence of let-7d-5p, miR-101-3p, and miR-133b as highly ranked miRNAs in expression, all of which commonly target 665 proteins. The most significant of these proteins play roles in focal adhesion, the P53 signalling pathway, and spectrin binding (Figure 6A). The relevance of focal adhesion and spectrin in cytoskeletal dynamics and barrier integrity has been well established in the literature, alongside their interaction that contributes to such. For instance, αII-spectrin (SPTAN1) is a crucial regulator of cell adhesion molecules and cellular shape changes [113]. While the paradoxical association between focal adhesion and p53 pathway, mediated by focal adhesion kinase (FAK/PTK2), is an interesting discussion in the tumour niche [114], such a cascade can result in the modulation of cell survival, substrate adhesion, and maintenance of the undifferentiated state of epithelial cells through the Mouse Double Minute 2 homolog (MDM2) protein [115]. This presents a potentially coherent triad contributing to tight junction proteins redistribution [116], cytoskeletal integrity, and cell–cell adhesion [117,118]. Thus, one speculative model is that recurrently identified lean meat-associated miRNAs could intersect with mechanotransduction networks linking the extracellular matrix to cytoskeletal architecture. In this hypothetical framework, spectrin–ankyrin complexes (e.g., ANK3) and focal adhesion components could represent nodal points through which regulatory effects might converge, potentially influencing epithelial structural stability [119]. Additionally, cross-talk between focal adhesion signalling and p53-related pathways via mediators such as MDM2 or RHOA [115,120], which responds by regulating epithelial identity preservation by promoting the expression of epithelial-specific genes, e.g., E-cadherin (CDH1) and preventing EMT [121], has been described in epithelial identity regulation, suggesting a theoretical axis through which such interactions could occur.

#### 3.3.2. Meat Fat

Meat fat consistently highly ranked miRNAs (let-7i-5p, miR-30c-5p, and miR-23a-3p), exclusively targeted 2139 genes, the most significant of which play roles in Shigellosis, Dyenin complex, and HOPS complex (Figure 6B). One important interaction in the Shigellosis cluster is a six-protein interaction (SSH1, SSH2, PPP3CA, PPP3CB, PPP3R1, and NFATC3), which contributes to the calcineurin-nuclear factor of the activated T-cells (NFAT) signalling cascade. The calcineurin-NFAT signalling is an integral component of both the adaptive and innate immune responses in B and T lymphocytes [122]. Particularly, NFATC3 constitutes the toll-like receptor-activated innate inflammatory response and cytokine induction [123] and modulates nitric oxide (NO) release in macrophages to ensure bacterial clearance during infection [124]. Hence, the blockage of this cascade may be vital in inflammatory control [125]. In addition, the HOPS complex—a central hub for phago-some maturation, coordinating Rab7-dependent tethering, phagosomal acidification, and lysosomal enzyme delivery—is essential for microbial killing of internalised pathogens in phagocytic cells. Bacteria can use their hijacking to delay endosome maturation and ensure their survival in the host [126]. Another mode of control over inflammatory response is through the epidermal growth factor receptor (EGFR) signalling. The EGFR signalling induces nuclear factor kappa-B (NF-κB) and mitogen-activated protein kinase (MAPK1/3) pathways to induce cytokine and NO production and macrophage activation during bacterial infection, which results in chronic inflammation [127]. The signalling is deactivated through the degradation fate of EGFR trafficking, mediated by the HOPS complex. Hence, this trafficking route can be altered through the inhibition of the HOPS complex or components that promote their activity, such as ezrin and radixin proteins [128], which also constitute the shigellosis cluster. Additionally, the dyenin complex is an intracellular transport machinery employed by T-cells to transport microclusters to the centre of the immune synapse in a way that is inversely proportional to the cell activation [129]. A hypothetical immunoregulatory model here is that abundantly expressed meat fat-associated miRNAs could present a convergence with molecular nodes involved in inflammatory signalling, phagosome maturation, or immune synapse organisation. Such interaction may employ the modulation of components such as NFATC3, HOPS-associated proteins, or dynein-related transport machinery to influence immune signalling thresholds or pathogen-handling dynamics.

#### 3.3.3. Meat Offal

The highly ranked miRNAs particular to various meat offal documented in the literature (miR-145-5p, miR-92-5p, and miR-24-3p) have 244 shared validated targets. These proteins potentially constitute the actin cytoskeleton and TGF-β signalling pathway (Figure 6C). The modulation of inflammatory response via TGF-β1 upregulation by dietary extracellular vesicles and their miRNA cargoes, in a way that contributes to epithelial barrier integrity, has been demonstrated [130]. The TGF-B1 signalling can reduce the barrier disruption that can result from exposure to pathogens or pro-inflammatory cytokines [131]. This is achieved, amongst other routes, via the induction of the Smad signalling pathway components such as SMAD4 [132]. The interaction between the TGF-β signalling pathway and actin cytoskeleton has been previously reviewed [133]. Usually, such interactions are EMT-related and bidirectional [134,135,136]. Hence, while the signalling can induce cytoskeletal remodelling, resulting in cell shape alteration and tissue rigidity [137], the modulation of shared targets within the TGF-β–Smad axis and cytoskeletal regulators suggests an abstract intersection through which abundantly expressed miRNAs from meat offal might influence epithelial barrier-associated processes. Given the established crosstalk between cytoskeletal dynamics and TGF-β signalling, these pathways represent plausible nodes of convergence from a systems perspective.

#### 3.3.4. Dairy

**Figure 7:**
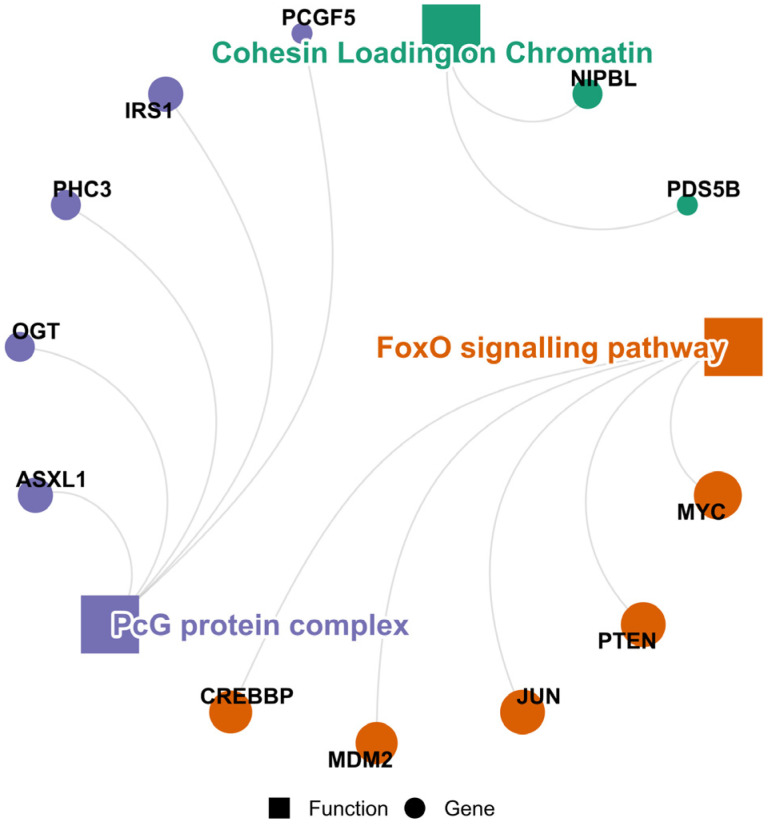
PPI cluster circular plots of the concordant/shared targets of dairy-restricted highly ranked and widely conserved miRNAs, indicating the hub proteins (top five) and the most significant functions (clusters) potentially modulated. Circle diameter is directly proportional to the node degrees.

The discriminatingly highly ranked miRNAs in dairy products (miR-200a-3p, miR-200c-3p, miR-223-3p, miR-25-3p, miR-29a-3p, and miR-29b-3p) commonly target 144 genes, clustered mainly into the FoxO signalling pathway and PcG protein complex (Figure 7). Melnik [138] proposed a working model of mTORC1 pronouncement, which involved the downregulation of the nuclear forkhead box transcription factor (FoxO) activity (extrusion from the nucleus) through the PI3K/AKT signalling. However, FoxO signalling promotes forkhead box (FoxP3) expression [139]. The FoxP3 is important for FoxP3^+^ regulatory T-cells (Tregs), which are linked to both dairy milk consumption in early life and a favourable immunoregulatory phenotype [140]. In essence, this relates to the maintenance of immune tolerance and homeostasis, while limiting excessive inflammation [141]. The main activity of the FoxO transcription factor family is in response to stress, and in so doing, maintains homeostasis, although they play a paradoxical role in tumour contexts [142]. In the latter context, modulation of FoxO by a member of the PcG protein complex (enhancer of zeste homolog 2) significantly induces EMT, tumour glycolysis, and cancer cell migration and invasion, specifically via FoxO1 downregulation [143]. However, for the most part, PcG protein complexes are crucial epigenetic regulators that silence gene transcription, maintaining cellular identity and controlling development by modifying chromatin to prevent unexpected developmental pathways [144]. Such cellular identity maintenance is vital to epithelial integrity, especially in the context of EMT. Taken together, this suggests a potential for immune tolerance, cell proliferation control and cellular identity by abundant, widely conserved miRNAs obtained dietetically from dairy products, presenting hypotheses for future studies.

#### 3.3.5. Seafood

**Figure 8:**
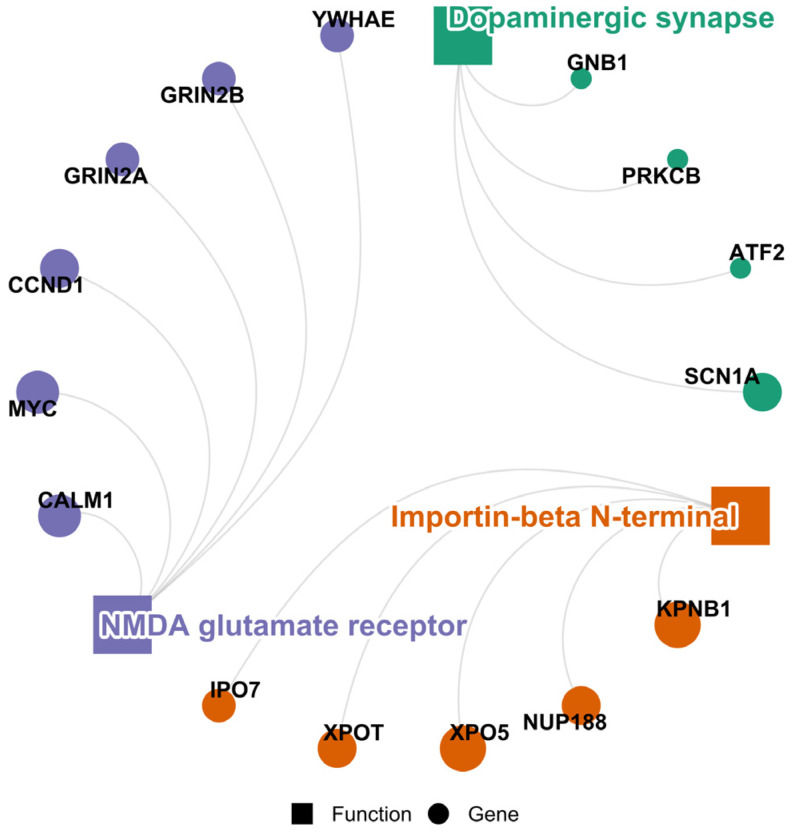
PPI cluster circular plots of the concordant/shared targets of seafood-restricted highly ranked and widely conserved miRNAs, indicating the hub proteins (top five) and the most significant functions (clusters) potentially modulated. Circle diameter is directly proportional to the node degrees.

Four widely conserved miRNAs (miR-17-5p, miR-184, miR-30e-5p, miR-92b-3p) are preferentially and consistently highly ranked in seafood, and they mainly target proteins involved in NMDA glutamate receptor and dopaminergic synapse (Figure 8). The N-methyl-D-aspartate (NMDA) glutamate signalling is a cascade employed by components of the central nervous system to produce stark effects based on context. Physiological cues are relayed by the signalling into neuronal survival and resistance, as well as synaptic plasticity, while the pathological environment is mediated into cell death through calcium ion (Ca^2+^) influx [145]. In this view, seafood consumption is linked to the NMDA glutamate receptor in two ways. The first is through marine-derived glutamate-like NMDA receptor agonists (such as domoic acid) and heavy metals accumulation in seafood products, the consumption of which could permit excitotoxicity and decreased NMDA receptor levels [146,147]. On the other hand, omega-3 fatty acids from seafood have been demonstrated to exert neuroprotective effects that could be a result of excessive NMDA receptor activation [148]. Given the possible components that come along with seafood intake, which are crucial for neural health, its highly ranked miRNA fraction may also be consistent with pathways involved in regulatory function in neurological contexts, through the regulation of its subunits (Glutamate receptor subunit epsilon-2A [GRIN2A] and GRIN2B) or dopaminergic interaction [149].

### 3.4. Documented In Vivo Evidence of Biological Activity of miRNAs from Animal-Source Foods in the Literature

Despite the back-and-forth regarding bioavailability and biological efficacy that ushered in the field of dietary miRNA [150,151,152,153], more recent studies continue to affirm the biological potential of food miRNAs, especially from milk, in in vitro settings to exert immunomodulatory, cell proliferation, and tissue repair potencies [154,155]. For instance, miR-27b from milk, which is also highly expressed in many animal-source foods, could promote apoptosis in colorectal cancer cells by increasing endoplasmic reticulum stress [156]. Similarly, Pieters et al. [157] suggested that milk miR-148a improves osteoarthritis prognosis and chondrocyte homeostasis by targeting DNA methyltransferase 3A. Chicken egg-derived nanovesicles and their miRNA cargoes were equally demonstrated to be internalisable and promote cell survival in porcine embryos [158,159]. Of particular relevance here, the regulation of epithelial barrier function by dietary miRNAs, mainly from dairy products, has been adequately documented [160,161,162]. This is often attributed to the decrease in proinflammatory cytokine production and increase of resistance against inflammatory pathways, such as NF-kB, by miRNAs [163,164]. For instance, miR-200a-3p (one of the selectively abundant miRNAs in dairy products) is of interest here, being associated with intestinal inflammatory control in mouse models [165].

A major number of studies investigating the biological activity of animal-sourced food-derived miRNAs in animal models focused on the use of milk. This may be partly due to milk being the pioneer food product of dietary miRNA study [166,167,168]. Additionally, being in liquid form, extraction protocols for miRNA isolation from milk are more simplified than other foods of a higher consistency, which usually require more preprocessing steps [169,170]. The ability to survive digestion, bioavailability, and therapeutic exploitation of milk-derived exosomes in murine models of colitis has been recorded [130,171]. Since EVs are potent shuttlers of genetic materials, including miRNAs [172,173], their biological activity is also usually attributed to their cargoes. Hence, the ultrasonication of milk, which exposes the contained miRNA to degrading RNase, is associated with a loss of the observed exosomal biological activity [165,174].

miRNA-carrying exosomes in food have distinct distribution patterns following ingestion in animal models and may not stay in the bloodstream for long, primarily accumulating in the liver, intestine, and lungs [165,175]. Hence, Swanson et al. [67] could demonstrate the presence of only three bovine-specific milk miRNAs in the porcine plasma 6 h after consumption. Dietary miRNAs are far-reaching in their distribution, and the abundantly expressed ones could pass through even the placenta and the blood–brain barrier [165,175,176] and exert biological activity. Hence, their absence or deficiency could manifest at those distant target sites. Therefore, the deficiency of RNA in eggs fed to murine models was enough to cause cognitive impairment [158]. Although the abundance of complementary sites of mRNA targets may drive miRNA accumulation, what governs the extent of miRNA distribution is unknown.

Depending on the context of the disease, dietary patterns can alter circulating miRNA expression profiles in humans [177,178]. More specifically, the inclusion of meat and milk in the diet was associated with the upregulation of selected miRNAs involved in cell cycle progression, β-cell function, and insulin sensitivity, including miR-17, miR-21, miR-122-5p, and miR-223-3p [178,179,180]. Although dietary miRNAs are demonstrated to be bioavailable for absorption following digestion in human subjects, there are still uncertainties regarding the biological relevance of the consumed dose in the dietary matrix [158,181,182]. Indeed, there are only a few studies inquiring into dietary miRNA transference from foods of animal sources into human-based models, perhaps due to the challenging nature of setting up such an experimental design.

## 4. Research Outlook

Several food types lack representation in this study due to their limited coverage in the literature. Poultry products are a case in point: although there are studies examining tissues derived from poultry, such samples fall outside our definition of food products here. Additionally, to denote a food product as a source of dietary miRNAs, the digestive stability of the contained miRNAs needs to be ascertained. Knowing that miRNAs exhibit differential stability under gastrointestinal digestive conditions [112], as many dietary sources as possible need to be screened in this regard. Aside from milk, the available documentation of ASF miRNA behaviour under gastrointestinal conditions is rather limited. Future studies investigating dietary miRNA biological activity will benefit immensely from such a basis, as they will provide essential insights into the potential for dietary miRNAs to exert physiological effects post-ingestion.

The predicted targets of the identified consistently highly ranked and ultra-conserved dietary miRNAs from animal sources in this study portrayed important roles in epithelial barrier integrity, and this might be an interesting area for dietary miRNAs. Since the deprivation of RNAs in animal-sourced foods has been suggested as a potent factor influencing cognitive abilities in a murine model [158], the potential of ingested miRNAs, particularly from seafood, in neural health deserves more investigation. Furthermore, since the miRNA profile of animal-sourced food products may affect their quality and play a potential role in oral tolerance development in humans [68,163,180,181,182,183], investigations into the relationship between miRNA levels in food and food choice, selection, and overall acceptance will offer an interesting view of how such nucleic acids promote their continually increased intake.

## 5. Conclusions

Here, we compiled evidence supporting the presence of 48 highly conserved miRNAs, consistently ranking among the top within studies of animal-sourced foods and analysed their possible targets. Enrichment analysis indicated overrepresentation of pathways related to epithelial barrier integrity. While lean meat and offal, through their predominant miRNA fraction, may align more to pathways related to these processes, meat fat and dairy may be more associated with inflammatory control, and seafood, with glutamate signalling. This stipulates the potential of animal-sourced foods to offer both epithelial and immune barrier protection by their miRNA fraction. However, because target prediction and pathway enrichment rely on computational algorithms and the measure of miRNA predominance employed here is a cross-study recurrence of high relative abundance, the biological interpretations presented here should be considered exploratory. Demonstrating functional activity of dietary miRNAs in vivo will require controlled experimental validation. The literature base for this study, which was limited to ruminant livestock and their milk products, presents the need for a broad view of animal-sourced foods. In light of this, future studies will immensely benefit from a comprehensive conserved miRNA profiling of a wider range of such foods. A prerequisite for the biological efficacy of food-contained biomolecules, such as miRNAs, is their gastrointestinal stability information, which is lacking in the literature. Though experimental studies demonstrating the activities of these miRNAs are rather limited to milk and poultry products, the enrichment of targets for highly expressed miRNAs in animal-sourced foods in epithelial barrier integrity may offer a riveting view of these food biomolecules.

## Data Availability

The original contributions presented in this study are included in the article and supplementary materials. Further inquiries can be directed to the corresponding author.

## Supplementary Materials

The following supporting information can be downloaded at: https://www.mdpi.com/article/10.3390/cimb48020237/s1.

## Abbreviations

The following abbreviations are used in this manuscript:

ASF	Animal-sourced food
ANK3	Ankyrin 3/G
CCND1	Cyclin D1
CDC42	Cell division cycle 42
CDH1	Cadherin 1
DCC	Deleted in colorectal cancer
ECM	Extracellular matrix
EGFR	Epidermal growth factor receptor
EMT	Endothelial mesenchymal transition
FAK	Focal adhesion kinase
FoxO	Forkhead box, class O
FYN	Tyrosine-protein kinase Fyn
GRIN2	Glutamate Ionotropic Receptor NMDA Type (or epsilon) Subunit 2
HOPS	Homotypic fusion and vacuole protein sorting)
IgSF CAM	Immunoglobulin superfamily cell adhesion molecule
KEGG	Kyoto Encyclopedia of Genes and Genomes
MAPK	Mitogen-activated protein kinase
MDM2	Mouse double minus 2
miRNA	microRNA
mTORC1	Mammalian Target of Rapamycin Complex 1
MYC	Myelocytomatosis proto-oncogene
NFATC3	Nuclear Factor of Activated T-cells, cytoplasmic 3
NF-κB	Nuclear Factor kappa-light-chain-enhancer of activated B cells
NMDA	N-methyl-D-aspartate receptor
NO	Nitric oxide
PcG	Polycomb group protein
PPP3CA	Protein Phosphatase 3 Catalytic Subunit Alpha.
PPP3CB	Protein Phosphatase 3 Catalytic Subunit Beta.
PPP3R1	Protein Phosphatase 3 Regulatory Subunit B, Alpha
PTK2	Protein Tyrosine Kinase 2 or Focal adhesion kinase (FAK)
RHOA	Ras homolog family member A
SMAD4	Mothers against Decapentaplegic Homolog 4
SPTAN1	Spectrin Alpha, Non-Erythrocytic 1
SSH1	Slingshot Protein Phosphatase 1
SSH2	Slingshot Protein Phosphatase 2
TGF-β	Transforming growth factor beta
UTR	Untranslated region
