# Supplementary material for: Predominant miRNAs in Animal-Source Foods and Bioinformatic Analysis
Source: Curr Issues Mol Biol. 2026 Feb 23;48(2):237. doi: 10.3390/cimb48020237 (PMC12939316; doi:10.3390/cimb48020237)
Supplement: Supplementary file 1 [file cimb-48-00237-s001.zip › Supplementary Figures (1).pdf]

Supplementary Figures

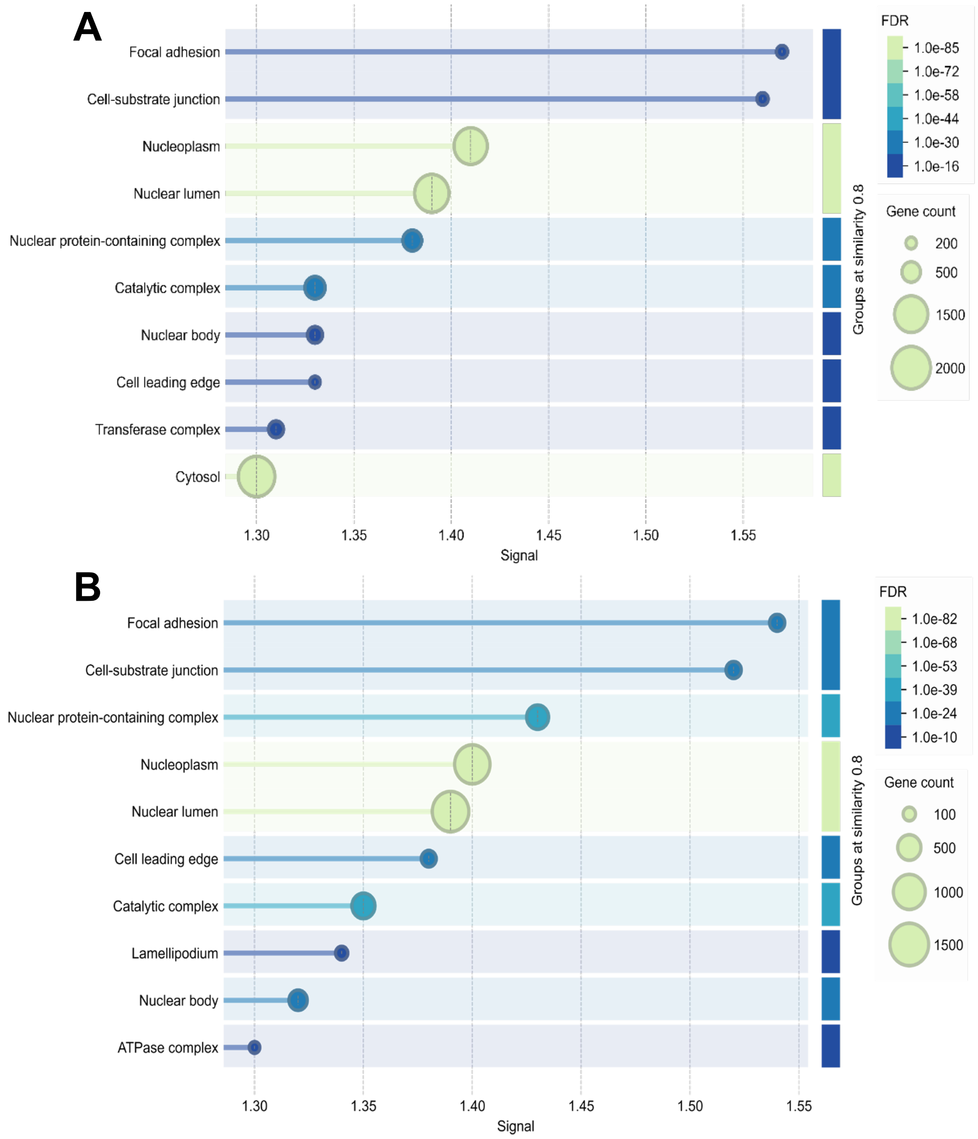

Figure S1. GO cellular compartment enrichment of targets of predominantly-expressed, broadly conserved miRNAs in foods of animal sources through KEGG pathway analysis of raw (**A**) and processed (**B**). Plotted using the STRING tool.

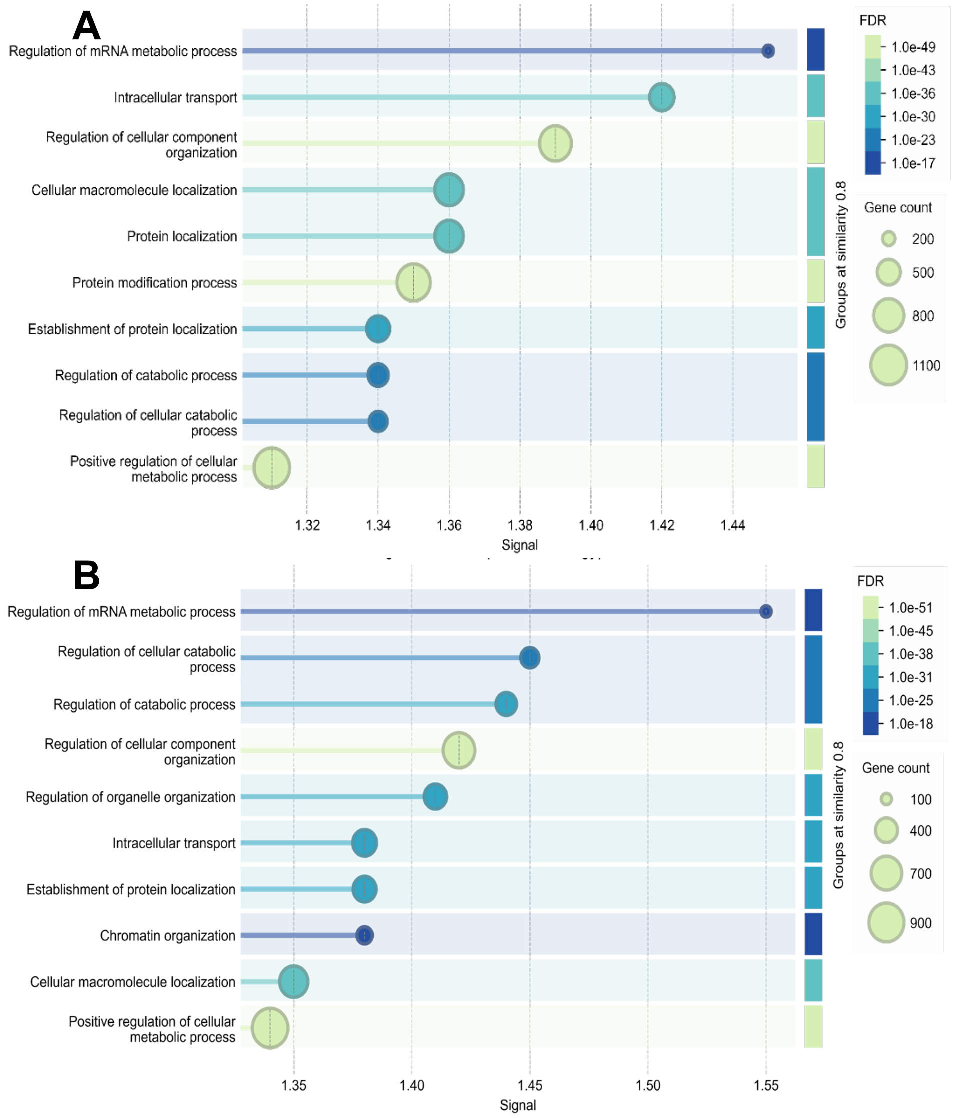

Figure S2. GO biological process enrichment of targets of predominantly-expressed, broadly conserved miRNAs in foods of animal sources through KEGG pathway analysis of raw (**A**) and processed (**B**). Plotted using the STRING tool.

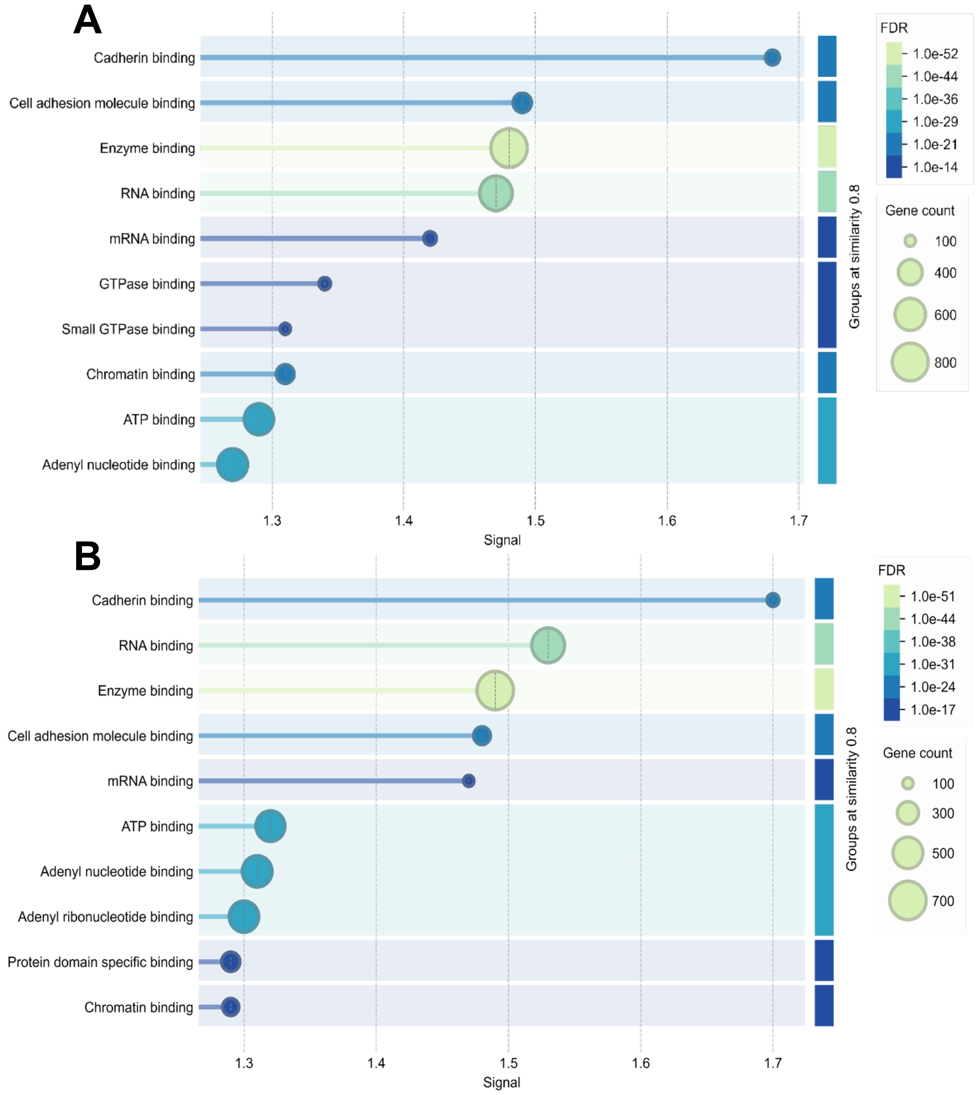

Figure S3. GO molecular function enrichment of targets of predominantly-expressed, broadly conserved miRNAs in foods of animal sources through KEGG pathway analysis of raw (**A**) and processed (**B**). Plotted using the STRING tool.
